# Integrated phenotypic analysis, predictive modeling, and identification of novel trait-associated loci in a diverse *Theobroma cacao* collection

**DOI:** 10.1186/s12870-025-07128-y

**Published:** 2025-08-09

**Authors:** Insuck Baek, Minhyeok Cha, Seunghyun Lim, Brian M. Irish, Sookyung Oh, Jishnu Bhatt, Rakesh K. Upadhyay, Moon S. Kim, Lyndel W. Meinhardt, Sunchung Park, Ezekiel Ahn

**Affiliations:** 1 Environmental Microbial and Food Safety Laboratory, Agricultural Research Service, Department of Agriculture, Beltsville, MD 20705 USA; 2 Sustainable Perennial Crops Laboratory, Agricultural Research Service, Department of Agriculture, Beltsville, MD 20705 USA; 3 Plant Germplasm Introduction and Testing Research Unit, Agricultural Research Service, Department of Agriculture, Prosser, WA 99164 USA; 4 Department of Natural Sciences, College of Arts and Sciences, Bowie State University, 14000 Jericho Park Rd., Bowie, MD 20715 USA

## Abstract

**Background:**

Cacao (*Theobroma cacao* L.) breeding and improvement rely on understanding germplasm diversity and trait architecture. This study characterized a cacao collection (173 accessions) evaluated in Puerto Rico, examining phenotypic diversity, trait interrelationships, and performing comparative analyses with published Trinidad and Colombia datasets. We also developed machine learning (ML) models for yield prediction and identified yield-associated SNP markers.

**Results:**

The cacao collection showed significant phenotypic variation and strong intra-collection trait correlations. Comparative analyses revealed conserved trait responses across environments, notably linking susceptibility to black pod rot in Puerto Rico with Witches' Broom Disease in Colombia, suggesting a broad-spectrum disease response mechanism. Machine learning models effectively modeled yield, quantifying a hierarchy of predictor importance, with ‘Total pods’, ‘Infection rate’, and ‘Pod weight’ being the most influential. Integrating existing SNP data for 28 common accessions, multiple SNPs were identified as significantly associated with key horticultural traits, including ‘Total pods’, ‘Infection rate’, and ‘Yield’ (FDR < 0.01). Notably, a single genetic marker on chromosome 5 (TcSNP475), located within a putative zinc finger stress-associated protein gene (*Tc05_t008610*), was associated with both ‘Total pods’ and ‘Yield’, representing a prime target for marker-assisted selection.

**Conclusions:**

This research provides a detailed characterization of a wide germplasm collection, robust yield predictors, and a suite of novel trait-linked genetic markers, offering valuable resources for cacao breeding. These integrated findings will provide a solid foundation for targeted breeding strategies and deeper molecular investigations into the mechanisms underpinning yield and stress resilience in this vital global crop.

**Supplementary Information:**

The online version contains supplementary material available at 10.1186/s12870-025-07128-y.

## Introduction

*Theobroma cacao* L., the sole source of raw beans (i.e., cocoa beans/seeds) for the multi-billion dollar global chocolate industry, is a cornerstone of tropical agriculture, supporting the livelihoods of millions of smallholder farmers worldwide [1]. As a diploid (2n = 2x = 20) tree species native to the tropical rainforests of South America with a genome size of approximately 430 Mb, its cultivation is now global [2]. As a cauliflorous species that flowers directly on its trunk and branches and relies on insect pollination [3, 4], its cultivation is concentrated in specific tropical regions. Global production is approximately 4.5 million tonnes annually, with West African countries such as Côte d’Ivoire and Ghana supplying over 60% of the total volume [5, 6]. In Colombia, a major producer, the average yield is close to 400 kg/ha, or 0.4 tons per hectare [7]. The enduring demand for high-quality cocoa products, alongside significant production challenges such as pests, diseases, and unfavorable weather conditions, necessitates continuous efforts in cacao breeding programs. These programs aim to enhance yield by bolstering resistance to prevalent diseases, insect pests, and environmental stresses, while improving the quality of beans and chocolate [1, 8]. Central to such improvement efforts is a profound understanding of the rich, albeit often underutilized, genetic diversity harbored within cacao germplasm collection [9, 10] and the complex interplay of horticultural traits that determine overall productivity and disease response. Indeed, comprehensive phenotypic characterization of existing germplasm collections provides the essential foundational data for identifying superior genotypes and dissecting trait architecture, a critical first step in effective breeding strategies [8, 11, 12].

While small-scale germplasm evaluations yield valuable site-specific information, integrating data across larger and more diverse genetic pools and environments can provide broader insights into trait stability, genotype-by-environment (G×E) interactions, and the identification of more universally valuable genetic resources. This addresses a critical aspect given the challenges G×E interaction poses to breeding progress in perennial crops [13–15]. Modern analytical techniques, including multivariate statistics and machine learning (ML), offer powerful tools to dissect complex phenotypic datasets, reveal underlying population structures, elucidate trait interrelationships, and develop predictive models for key performance indicators such as yield, as increasingly demonstrated in cacao and other crops [11, 16]. Such predictive capabilities can significantly streamline the breeding program selection process, which is particularly beneficial for perennial tree crops like cacao with long generation cycles [8].

Alongside robust phenotypic assessment, the integration of molecular data, particularly Single Nucleotide Polymorphisms (SNPs), has significantly advanced crop improvement strategies by providing powerful tools for genetic analysis and targeted breeding [17, 18]. In cacao, genome-wide association studies (GWAS) and similar approaches are increasingly utilized to identify genetic loci linked to crucial horticultural traits, including yield components and disease resistance [9, 11, 19]. Such discoveries pave the way for marker-assisted selection (MAS) and enhance the precision of breeding programs [8, 17]. The subsequent identification of candidate genes underlying these marker-trait associations is critical, as it can illuminate the biological pathways controlling complex traits and lead to the development of functional molecular markers or precise targets for advanced breeding techniques [11, 18]. Despite this progress, a comprehensive understanding of the genetic basis of many complex traits in *T. cacao*, particularly within diverse germplasm evaluated across varied regional conditions, different disease pressures, and environmental responses, remains an active area of research. This underscores the ongoing need for integrated genetic studies and thorough germplasm characterization to fully harness cacao’s genetic potential [8, 10].

This research undertakes a multi-faceted approach to characterize a diverse cacao collection (173 accessions) evaluated in Puerto Rico (PR). Our objectives were to: (1) characterize the phenotypic diversity and structure of the collection by evaluating key horticultural traits and their interrelationships using multivariate statistical analyses; (2) conduct comparative analyses with published datasets to identify conserved trait relationships and environmental specificities; (3) develop and evaluate ML models for predicting cacao yield based on phenotypic traits; and (4) identify potential SNP markers associated with key horticultural traits and explore linked candidate genes. Through this integrated approach, we aim to provide valuable insights into the horticultural potential of the studied germplasm collection, identify robust predictive tools for cacao breeding, and pinpoint a suite of genetic markers that could contribute to the development of improved cacao varieties.

## Materials and methods

### Plant material and phenotypic data evaluated in Puerto Rico

The primary phenotypic dataset utilized in this study originated from the USDA-ARS Tropical Agriculture Research Station (TARS) and National Plant Germplasm System’s *T. cacao* collection, located at the USDA-ARS Tropical Agriculture Research Station (2200 Pedro Albizu Campos Avenue, Suite 201, Mayagüez, Puerto Rico 00680–5470). This valuable historical dataset, collected from 2007–2011 for germplasm characterization, was recently compiled and integrated with modern genomic and analytical resources for the comprehensive analysis presented herein. Data were collected over a five-year period (2007–2011) for 173 cacao accessions. The experimental design employed a randomized complete block design with three blocks, where each accession was represented by two trees per block, resulting in a total of six trees per accession. In some cases, fewer than six trees were available due to plant loss. All trees were grafted onto ‘Amelonado’ rootstock, selected for its homozygosity, and maintained under full sun. Standard management practices included regular pruning to maintain a low canopy (~ 2.5 m), irrigation as needed during dry months (January-April), granular fertilizer application (15-5-10-3 with minor elements) every four months, and herbicide and insecticide applications as needed. The soil at the experimental site is a highly weathered, acidic Consumo series clay. The climate in Mayagüez during the 2007–2011 evaluation period was tropical, with an average annual temperature of approximately 26.2°C and an average annual rainfall of approximately 5151 mm (NOAA National Centers for Environmental Information). Trees were planted in a diamond pattern, with two meters between trees and rows, and a three-meter alley between every second row for equipment access. Evaluations commenced approximately six years after field establishment. For fruit- and seed-based traits, a representative sample of up to 50 healthy, average-sized pods was collected for each accession over the evaluation period. All seeds from these sampled pods were then used for downstream analyses. Phenotypic traits collection included seed characteristics such as total fresh seed weight per pod (g), total dry seed weight per pod (g) (after micro-fermentation and drying to ~ 7% moisture), number per pod, and seed water content (g, calculated as fresh weight − dry weight). Pod characteristics included length (cm), width (cm), weight (g), and index (number of pods required to yield one kilogram of dry beans). Yield components assessed were ‘Total pods’ per tree and ‘Yield’ (estimated as kilograms of dry beans per tree per year). Additionally, disease incidence, primarily from black pod rot (*Phytophthora* spp.), was recorded as the ‘Number of infected pods’ per tree and ‘Infection rate’ (calculated as the number of infected pods divided by total pods per tree). Detailed characterization data and passport information for the cacao accessions are publicly available through the USDA-ARS Germplasm Resources Information Network (GRIN)-Global database. The specific evaluation dataset is documented under the group name “CACAO.MAYAGUEZ.2007–2011”, with further details accessible via GRIN-Global method ID 495,345 (https://npgsweb.ars-grin.gov/gringlobal/method? id=495345 ).

All statistical analyses were performed using JMP Pro 17 (SAS Institute Inc., Cary, NC, USA) [20]. To further explore the overall structure within the TARS (PR) phenotypic dataset, PCA was performed using all measured horticultural traits for the 173 accessions. The contributions of individual traits to the principal components were examined using loading plots. Hierarchical clustering, using Ward’s method [21] with Euclidean distance, was applied to both the accessions (based on their complete phenotypic profiles) and the traits (based on their correlation patterns across accessions) to identify natural groupings. Pearson’s correlation coefficients (*r*) were calculated to assess the linear relationships between all pairs of measured horticultural traits within the primary set of 173 accessions evaluated in this study, with significance determined at *p* < 0.05. The correlation results were visualized using a matrix of scatter plots and a heatmap of correlation coefficients.

### Comparative analyses with published datasets

For comparative analyses, phenotypic and/or genotypic data were obtained from two published studies. The first was Bekele et al. [22], which provided phenotypic data for 27 flower, fruit, and seed traits, and SNP genotyping data (671 high-quality SNPs) for 421 cacao accessions conserved at the International Cocoa Genebank Trinidad (ICGT). The second was Osorio-Guarín et al. [7], which supplied phenotypic data related to productivity and disease resistance and over 8,000 SNP genotyping data for accessions from the Agrosavia germplasm collection in Colombia [7].

Lists of cacao accessions from the TARS dataset were compared against these published datasets to identify common accessions. The identities of the accessions in the primary USDA-ARS TARS collection were previously confirmed with microsatellite fingerprinting [23]. For the comparative analyses, the identification of common accessions between the TARS, ICGT, and Agrosavia datasets was based on the accession names and identifiers as reported in the respective source publications. We acknowledge that potential homonymy and synonymy across international genebanks is a known challenge. It is important to note that the three studies (TARS, ICGT, and Agrosavia) were designed and conducted independently. Consequently, the full sets of traits evaluated and the specific phenotyping protocols, including the number of trees evaluated per accession, were not standardized across locations. Our comparative analyses were therefore focused on the subset of traits that were directly or conceptually comparable across datasets, such as ‘Number of Seeds’, ‘Pod Index’, and general disease incidence.

After accounting for available data, 28 accessions were identified as common between the TARS evaluation and the Bekele et al. [22] SNP dataset and were used for the marker-trait association analysis. For direct phenotypic comparisons between the TARS and ICGT datasets, one of these accessions lacked phenotypic data in the ICGT collection, resulting in a set of 27 common accessions for those specific analyses. For the comparison with the Osorio-Guarín et al. [7] study, 20 common accessions were identified.

The three collections used for comparative analyses were maintained under different conditions and included trees of different ages. The primary TARS evaluation was conducted on mature trees that were approximately 6 to 10 years old during the 2007–2011 data collection period. For the Agrosavia, Colombia dataset, evaluations were performed from 2016 to 2018 on trees that were planted in 1998 (approximately 18–20 years old) [7]. The ICGT, Trinidad dataset was collected from established, mature trees, with phenotypic sampling occurring over an extended period between 1992 and 2012 [22].

Several analyses were subsequently performed on these overlapping subsets. For the 28 accessions common with Bekele et al. [22], each was assigned to one of the K = 7 genetic clusters based on its highest membership coefficient. Mean trait values were then compared across these clusters using Analysis of Variance (ANOVA) and post-hoc Student’s *t*-tests (*p* < 0.05). To explore the relationship between genetic structure and phenotype, Pearson’s correlation was used to compare membership coefficients with TARS phenotypic traits. To compare trait expression across environments, Spearman’s rank correlation coefficients (*ρ*) were calculated between the TARS collection traits and the ICGT dataset traits for these 28 accessions [22]. For the 20 accessions common with the Osorio-Guarín et al. study, Pearson’s correlation coefficients (*r*) were calculated between numerical traits from the TARS collection and the Agrosavia dataset [7].

### SNP data integration and marker-trait association analysis

The generation of this SNP panel, as detailed by Bekele et al. [22], involved selecting markers located in coding sequences with similarity to known proteins. Genotyping was performed using the Illumina BeadArray platform with the GoldenGate Assay. The final high-quality set of SNPs was established after genotype calls were verified against reference genotypes and the data was filtered for quality.

For the 28 accessions common to both the TARS phenotypic dataset and the Bekele et al. [22] SNP panel, a response screening analysis was performed in JMP Pro 17. Each of the 671 SNPs was tested as a predictor for each of the TARS phenotypic traits. The significance of associations was determined using an FDR (False Discovery Rate) correction, with a threshold of *p* < 0.01. Additionally, an analysis was conducted using over 8,000 SNP markers (referenced to Criollo and Matina genomes) from accessions in the Agrosavia germplasm collection (Colombia), matching them against the TARS phenotypic data [7]. However, this analysis did not yield any significant marker-trait associations for any of the traits examined.

For the SNPs found to be significantly associated with horticultural traits, potential candidate genes in proximity were identified using the *Theobroma cacao* Criollo v1/v2 reference genome assembly and annotations available through the CocoaGen DB genome browser (http://cocoa-genome-hub.southgreen.fr/jbrowse*).*

### Machine learning models for yield prediction

#### Model development and validation

The Model Screening function in JMP Pro 17 was used to develop and evaluate various ML models for predicting ‘Yield’ using the phenotypic dataset from the TARS evaluation. The models tested were selected to represent a diverse range of algorithmic approaches, facilitating a comprehensive comparison of their predictive performance for this specific application. These included: ensemble methods (Neural Boosted [24], Boosted Tree [25], Bootstrap Forest [26]), regression techniques (Fit Stepwise [27], Generalized Regression Lasso [28], Fit Least Squares [29]), Support Vector Machines (SVM with RBF kernel) [30], Decision Tree [31], and K-Nearest Neighbors (KNN) [32]. Model performance was assessed using 5-fold cross-validation (random seed = 1), with R-squared as the primary metric. The average R^2^ across the 5 validation folds and the R^2^ of the best individual validation fold were considered.

#### Iterative feature selection and predictor importance

An iterative modeling approach was employed. First, all available phenotypic traits from the TARS evaluation dataset (excluding ‘Yield’, which served as the target variable) were used as predictors. Second, the top predictor identified in the initial models (‘Total pods’) was removed, and the models were retrained. Third, the top predictor from this second iteration (‘Infection rate’) was also removed (in addition to ‘Total pods’), and the models were retrained again. For the best-performing model (Neural Boosted) in each iteration (specifically, from the best validation fold), the relative importance of the predictor variables was assessed based on their “total effect” values as reported by JMP Pro 17. Detailed results of model performance and predictor importance were compiled (Supplementary Data 1).

## Results

### Phenotypic diversity and trait interrelationships in the TARS collection

**Fig. 1:**
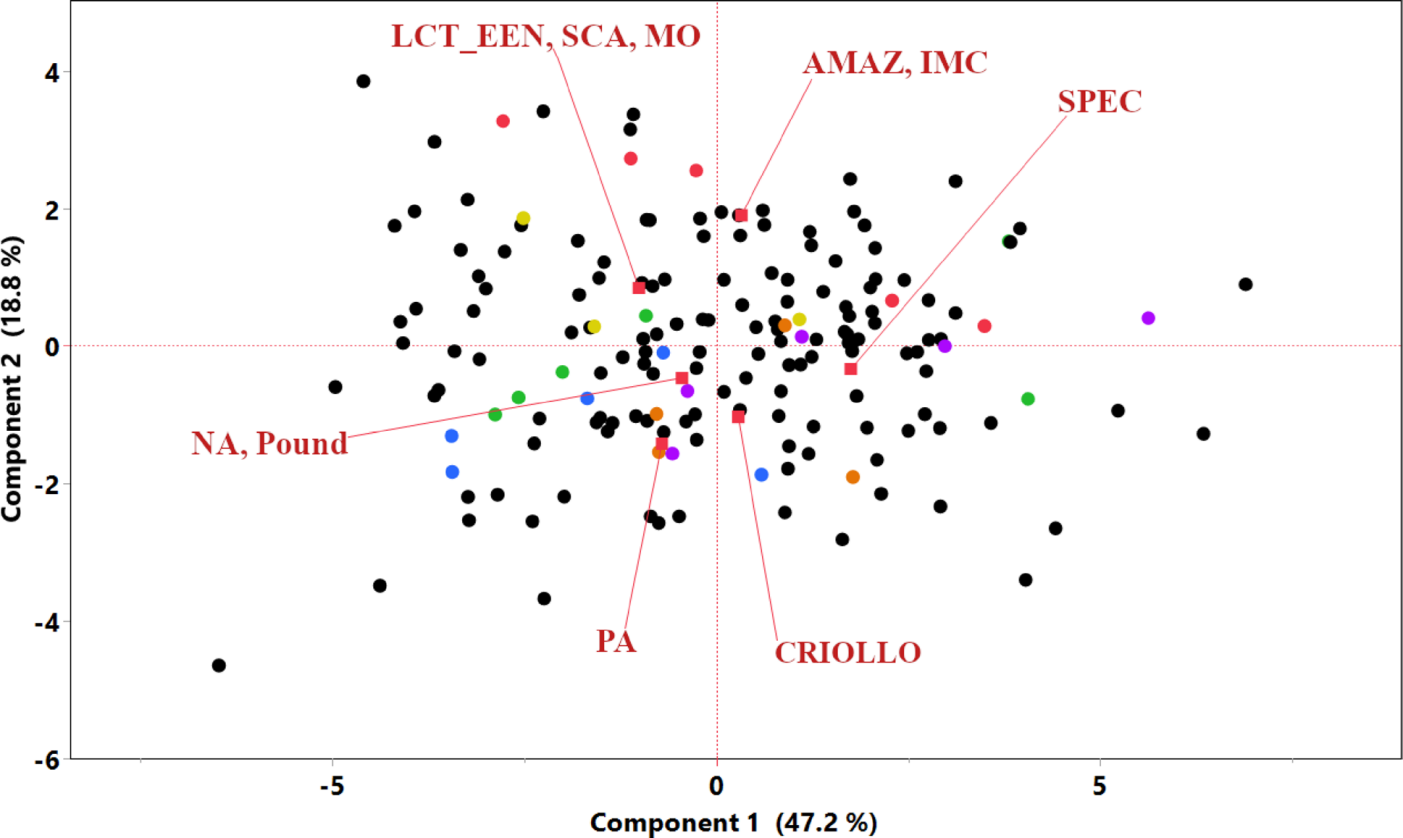
Principal component analysis of 173 cacao accessions based on all measured phenotypic traits. The plot displays the distribution of individual accessions along the first two principal components (PC1 and PC2). The 28 accessions with known genetic group assignments from Bekele et al. [22], are color-coded according to their respective group: AMAZ, IMC (red); CRIOLLO (orange); LCT_EEN, SCA, MO (yellow); NA, Pound (green); PA (blue); and SPEC (purple). The remaining 145 accessions are shown as black dots. Red vectors indicate the mean coordinates for each genetic group

To further explore the structure within the phenotypic data and the relationships among traits for the 173 accessions evaluated in TARS, PCA and Hierarchical Clustering were performed using all measured horticultural traits. The PCA revealed that the first principal component (PC1) accounted for 47.2% of the total phenotypic variance, while the second principal component (PC2) explained an additional 18.8%, collectively representing 66.0% of the variation. Figure 1 displays the distribution of all 173 individual accessions (black dots) in the PCA space based on their phenotypic profiles. The red labels represent the K = 7 genetic structure groups defined by Bekele et al. [22] and are positioned at the mean coordinates of the 28 common accessions belonging to each respective group. The mean points for the accessions show some differentiation. For instance, the means for the “NA, Pound” and “PA” Bekele groups are positioned in the lower-left quadrant, while the mean for “AMAZ, IMC” is in the upper-right quadrant, “LCT, EEN, SCA, MO” is in the upper-left quadrant, and “SPEC,” and “CRIOLLO” are in the lower-right quadrant. This visualization helps to position these genetically defined subsets within the broader phenotypic landscape of the entire TARS collection.

The contributions of individual traits to these principal components are illustrated in the loading plot (Fig. S1). Traits such as pod width, pod length, pod weight, seed water content, and fresh seed weight loaded heavily on PC1, indicating their primary role in driving the main axis of variation. Both PC1 and PC2 influenced dry seed weight, pod index, and total pods, though their directional influence varied. PC2 predominantly influenced Yield (kg/tree/year), number of seeds, and infection rate. The number of infected pods showed a minimal impact, primarily along PC2.

Hierarchical clustering using Ward’s method, based on all phenotypic traits, grouped the 173 accessions into 13 distinct clusters (Fig. S2). This clustering reflects the overall phenotypic similarity among accessions. A corresponding hierarchical clustering of the traits themselves revealed their interrelationships across the dataset (Fig. S3). Notably, dry seed weight clustered closely with pod length. Fresh seed weight formed a cluster with seed water content, which was adjacent to a group containing pod weight and pod width. The number of infected pods and infection rate clustered together, and this pair was closely associated with pod index. Finally, total pods and Yield (kg/tree/year) formed a distinct group, which was linked to the number of seeds. These multivariate analyses provide insights into the underlying structure of phenotypic diversity and the correlational patterns among key horticultural traits in this cacao collection.

**Fig. 2:**
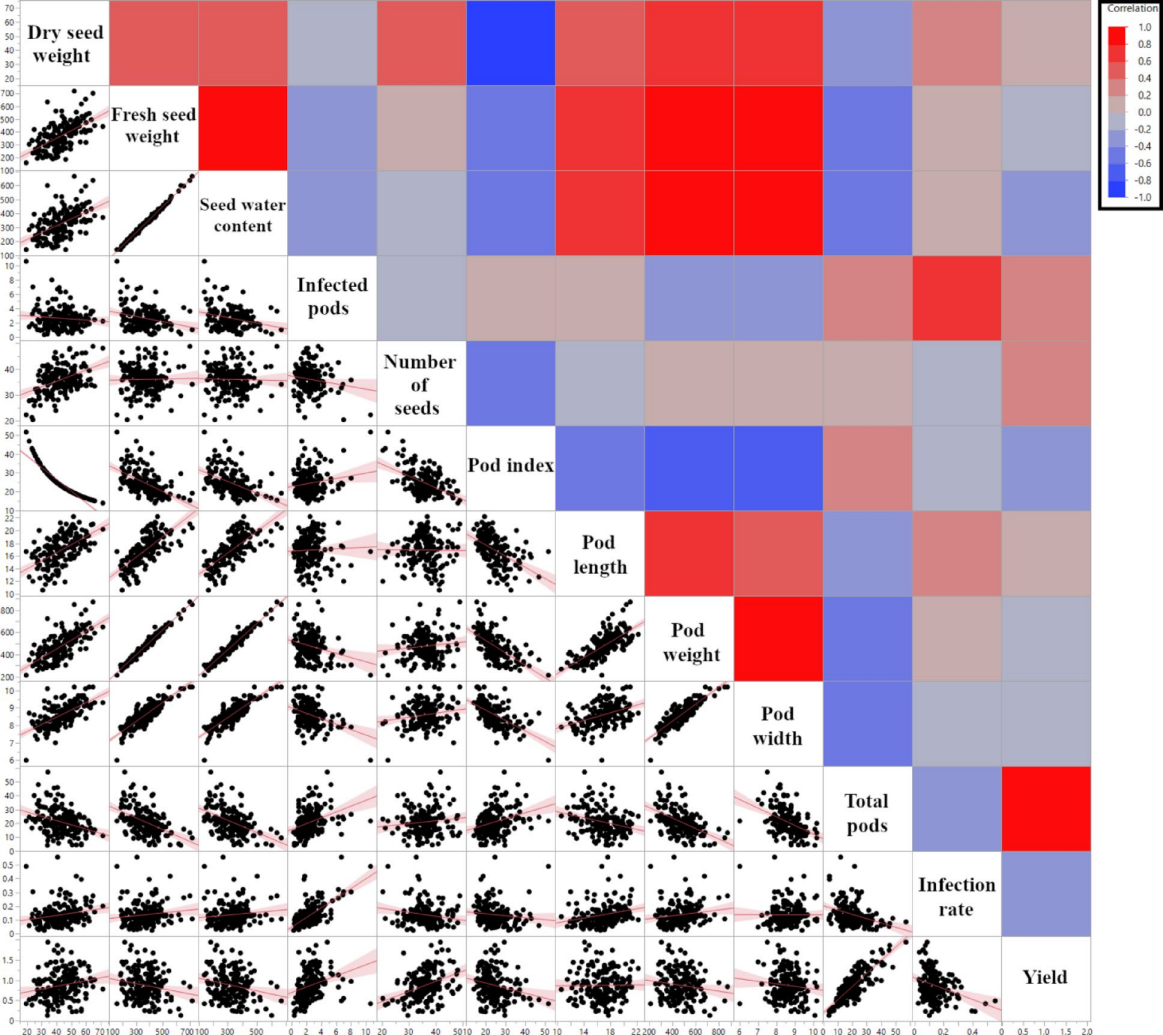
Pearson’s correlation matrix of horticultural traits from TARS cacao phenotype data. The upper triangle displays a heatmap of Pearson correlation coefficients (*r*), where red indicates positive correlations and blue indicates negative correlations. The lower triangle shows scatter plots for each pair of traits, with a fitted regression line. Trait names are indicated on the diagonal. Detailed correlation coefficients and *p*-values are provided in Supplementary Data 1

To understand the interrelationships among the horticultural traits measured in Mayaguez, PR, 2007–2011, and to explore phenotypic consistencies with other studies, several correlation analyses were performed: Pearson’s correlation analysis among all traits measured in the TARS collection revealed numerous significant associations, visualized as a heatmap and scatter plots in Fig. 2 (detailed *p*-values and correlation coefficients are provided in Supplementary Data 1). Notably, a strong positive correlation was observed between ‘Yield’ and ‘Total pods’ (*r* = 0.83, *p* < 0.0001). ‘Yield’ also showed a significant negative correlation with ‘Infection rate’ (*r* = − 0.26, *p* = 0.0006). The ‘Number of infected pods’ was strongly positively correlated with ‘Infection rate’ (*r* = 0.70, *p* < 0.0001). Strong positive correlations (often *r* > 0.70, *p* < 0.0001) were prevalent among pod size-related traits, including ‘Pod length’, ‘Pod weight’, and ‘Pod width’. Other notable strong correlations included ‘Dry seed weight’ with ‘Pod index’ (*r* = − 0.95, *p* < 0.0001), ‘Fresh seed weight’ with ‘Pod weight’ (*r* = 0.99, *p* < 0.0001), and ‘Seed water content’ with ‘Pod weight’ (*r* = 0.97, *p* < 0.0001), suggesting that seed water content is a major contributor to pod weight. ‘Number of seeds’ per pod was positively correlated with the total ‘Dry seed weight’ per pod (*r* = 0.45, *p* < 0.0001); consequently, a higher number of seeds was also strongly and negatively correlated with ‘Pod index’ (*r* = − 0.50, *p* < 0.0001), as fewer of these productive pods were required to yield one kilogram of dry beans. ‘Pod weight’ showed a significant negative correlation with the ‘Number of infected pods’ (*r* = − 0.23, *p* = 0.0027). ‘Total pods’ exhibited significant negative correlations with ‘Dry seed weight’ (*r* = − 0.32, *p* < 0.0001), ‘Seed water content’ (*r* = − 0.46, *p* < 0.0001), ‘Pod weight’ (*r* = − 0.48, *p* < 0.0001), and ‘Infection rate’ (*r* = − 0.32, *p* < 0.0001), the latter suggesting that higher pod production be associated with a lower overall infection rate.

### Comparative analyses of TARS traits with ICGT and Agrosavia collections

**Fig. 3:**
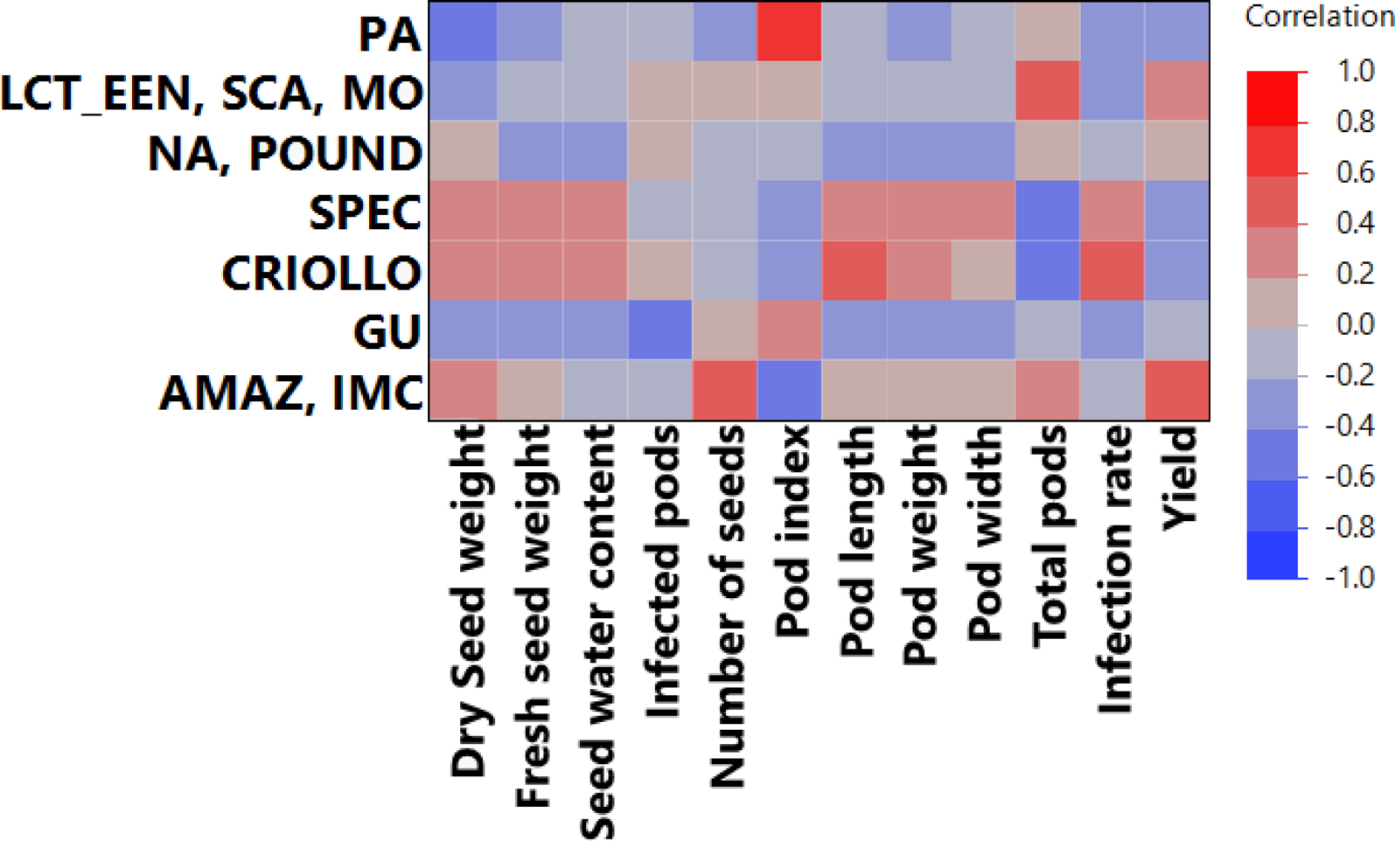
Correlation heatmap of genetic cluster membership and phenotypic traits. The heatmap displays Pearson’s correlation coefficients (*r*) between membership coefficients for 28 common accessions to the K = 7 genetic clusters defined by Bekele et al. [22] and the corresponding phenotypic traits evaluated in Puerto Rico. Red squares indicate positive correlations and blue squares indicate negative correlations, with color intensity corresponding to the magnitude of the correlation coefficient

To explore potential associations between genetic structure and horticultural traits in a subset of the cacao collection [23], Pearson’s correlation coefficients were calculated. This analysis involved 28 accessions common to TARS study and the Bekele et al. [22] investigation. Membership coefficients for these accessions to the K = 7 genetic clusters defined by Bekele et al. [22] were correlated with their phenotypic trait values from the TARS evaluation. Several statistically significant correlations were identified (Fig. 3, detailed in Supplementary Data 1). Notably, a higher membership coefficient for the ‘AMAZ, IMC’ genetic cluster was positively correlated with both the Dry seed weight (*r* = 0.39, *p* = 0.041), Number of Seeds per pod (*r* = 0.59, *p* = 0.001) and overall Yield (kg/tree/year) (*r* = 0.50, *p* = 0.0065), while being negatively correlated with Pod Index (*r* = − 0.40, *p* = 0.0344). Membership in the ‘LCT, EEN, SCA, MO’ cluster showed a positive correlation with Total Pods per tree (*r* = 0.48, *p* = 0.0097), whereas membership in the ‘SPEC’ cluster was negatively correlated with this trait (*r* = − 0.48, *p* = 0.0101). The ‘CRIOLLO’ genetic group membership showed positive correlations with Fresh seed weight (*r* = 0.38, *p* = 0.0454), seed water content (*r* = 0.38, *p* = 0.0475), Pod Length (*r* = 0.43, *p* = 0.0231) and Infection Rate (*r* = 0.50, *p* = 0.0074), and negatively correlated with total pods (*r* = − 0.41, *p* = 0.0318) Stronger affiliation with the ‘GU’ (Guiana) group was associated with fewer Infected Pods (*r* = − 0.40, *p* = 0.034). This correlation was possible because the analysis used continuous membership coefficients; however, the ‘GU’ group is not shown in Figs. 2 and 3, as no accession had ‘GU’ as its highest assigned ancestry group. Interestingly, membership in the ‘PA’ (Parinari) group was strongly correlated with a higher (less efficient) Pod Index (*r* = 0.61, *p* = 0.0005) and negatively correlated with Dry Seed Weight (*r* = − 0.48, *p* = 0.0098). These correlations suggest linkages between the genetic background and the expression of these specific horticultural traits within this subset of 28 accessions evaluated in Puerto Rico.

**Fig. 4:**
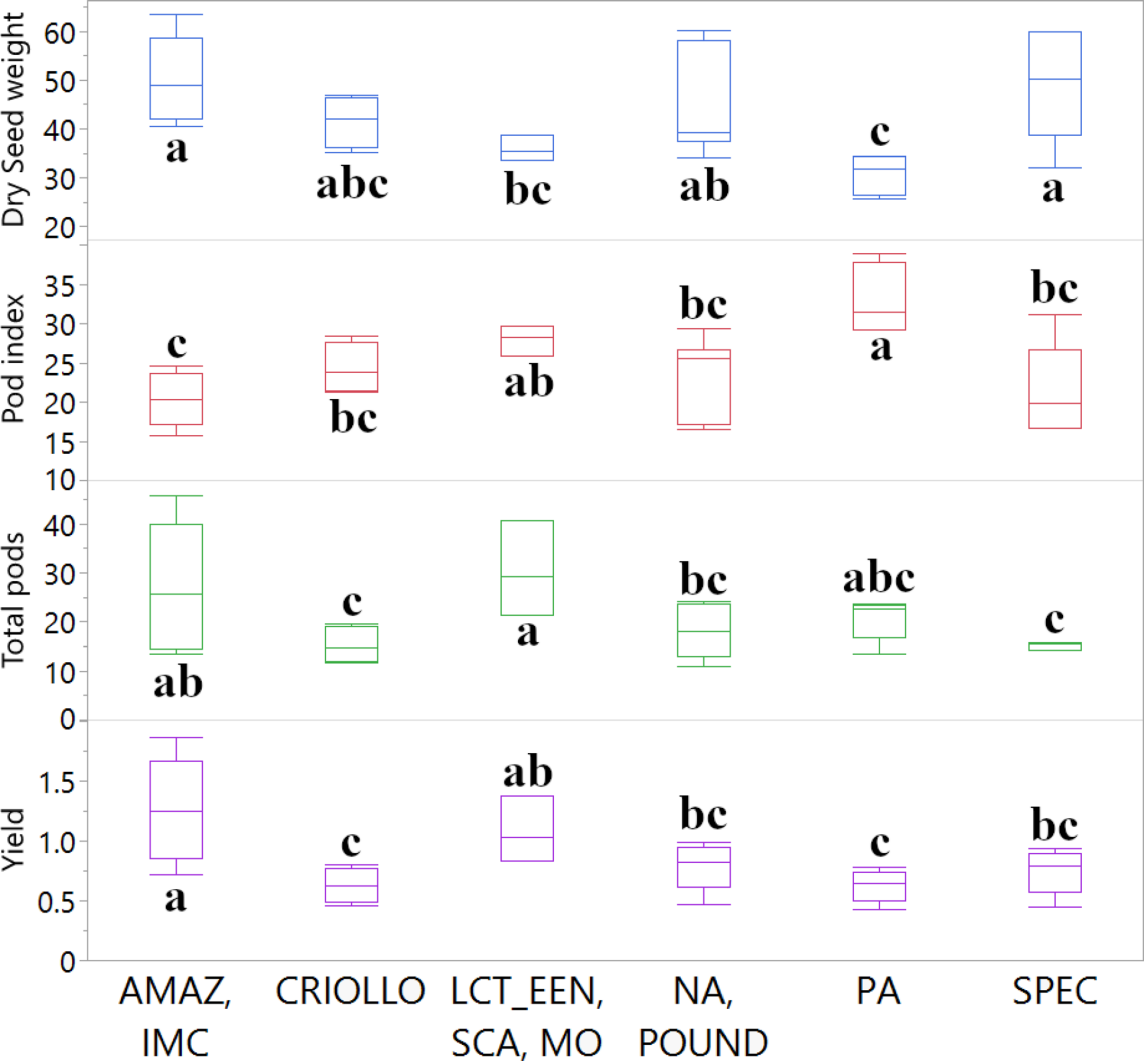
Comparison of key horticultural traits among 28 cacao accessions grouped by genetic clusters. Boxplots illustrate the distribution of Dry Seed weight (g), Pod index, Total pods (count), and Yield (kg/tree/year) for accessions assigned to the K = 7 genetic clusters. Other horticultural traits evaluated did not show statistically significant differences among these genetic groups in this subset of accessions. Boxes represent the interquartile range (IQR), the horizontal line within the box indicates the median, and whiskers extend to 1.5 times the IQR. Different letters above the boxes indicate statistically significant differences (*p* < 0.05) between mean values for the genetic groups, based on ANOVA and post-hoc Student’s *t*-tests

A summary of the ANOVA results and post-hoc pairwise comparisons (*p* < 0.05) for traits showing statistically significant differences among genetic groups is provided in Fig. 4. For Dry Seed Weight, the ANOVA revealed a statistically significant difference among the genetic structure groups (F = 3.7625, *p* = 0.0130). Post-hoc comparisons indicated that the ‘AMAZ, IMC’ group had a significantly higher mean dry seed weight (50.10 g) than the ‘PA’ (30.64 g) and ‘LCT, EEN, SCA, MO’ (35.83 g) groups. For Pod Index, a significant difference was also found among the groups (F = 5.3806, *p* = 0.0022). The ‘PA’ group had a significantly higher (less efficient) mean pod index (33.12) compared to all other groups except ‘LCT, EEN, SCA, MO’. Regarding yield components, the mean number of Total Pods per tree differed significantly among the genetic groups (F = 2.9832, *p* = 0.0333). The ‘LCT, EEN, SCA, MO’ group had a significantly higher mean number of total pods (30.43) than the ‘CRIOLLO’ (15.25), ‘SPEC’ (15.08), and ‘NA, POUND’ (18.05) groups. Finally, Yield (kg/tree/year) also showed a statistically significant difference among the groups (F = 4.7307, *p* = 0.0044). The ‘AMAZ, IMC’ group exhibited the highest mean yield (1.258 kg/tree/year), which was significantly greater than the ‘PA’ (0.626 kg/tree/year) and ‘CRIOLLO’ (0.630 kg/tree/year) groups. Other horticultural traits evaluated did not show statistically significant variation across these genetic groups for this subset of 28 accessions and are therefore not depicted in Fig. 4 (detailed statistics in Supplementary Data 1). These characterizations provide insights into the phenotypic diversity associated with the genetic structure within this subset of the cacao collection.

Spearman’s rank correlation was used to compare traits from the TARS study with those reported by Bekele et al. [22] for 27 overlapping cacao accessions evaluated at the ICGT. This analysis was chosen due to the inclusion of ranked and continuous data in the ICGT dataset. The correlation matrix is presented in Fig. S4 and Table 1, with full details available in Supplementary Data 1.

Evidence for trait stability across the two locations was observed. A significant positive correlation was found between ‘Number of seeds (TARS)’ and ‘Seed, number (ICGT)’ (*ρ* = 0.50, *p* = 0.0082), suggesting a degree of genetic control over this yield component. Similarly, ‘Dry weight (TARS)’ was strongly correlated with ‘Wet bean mass (ICGT)’ (*ρ* = 0.56, *p* = 0.0026), indicating consistency in the overall seed mass potential of these accessions.

The analysis also confirmed fundamental biological trade-offs. ‘Pod index (TARS)’, an inverse measure of efficiency, was strongly and negatively correlated with ‘Wet bean mass (ICGT)’ (*ρ*= −0.55, *p* = 0.0027). Conversely, ‘Total pods (TARS)’ showed a negative correlation with ‘Cotyledon mass (ICGT)’ (*ρ*= −0.48, *p* = 0.0118), demonstrating the classic inverse relationship between pod number and individual seed size.

Among the strongest relationships identified, ‘Pod length (TARS)’ was highly correlated with ‘Wet bean mass (ICGT)’ (*ρ* = 0.64, *p* = 0.0004), suggesting that the potential for longer pods is a strong indicator of the potential for heavier beans. Furthermore, an intriguing positive correlation was found between ‘Infection rate (TARS)’ and ‘Cotyledon mass (ICGT)’ (*ρ* = 0.44, *p* = 0.0205).

**Table 1:** **Significant spearman’s rank correlations between phenotypic traits from the Puerto Rico (**TARS**) and international cocoa Genebank**,** Trinidad (ICGT) evaluations.** The table lists significant (*p* < 0.05) spearman’s rank correlation coefficients (*ρ*) comparing traits measured in the 27 overlapping accessions from the TARS evaluation against traits from the ICGT dataset [22]. The corresponding *p*-value for each correlation is provided

Trait (TARS)	Trait (ICGT)	Spearman ρ	Prob>|ρ|
POD_LENGTH	Wet bean mass	0.6368	0.0004
POD_LENGTH	Pod index	−0.6298	0.0004
POD_LENGTH	Cotyledon length	0.585	0.0014
POD_LENGTH	Fruit, length	0.5801	0.0015
POD_LENGTH	Cotyledon mass	0.5602	0.0024
DRY_WEIGHT	Wet bean mass	0.5566	0.0026
POD_INDEX	Wet bean mass	−0.5543	0.0027
TOTAL_PODS	Pod index	0.5395	0.0037
NUMBER_OF_SEEDS	Fruit, basal constriction	−0.5331	0.0042
DRY_WEIGHT	Pod index	−0.5242	0.005
POD_INDEX	Pod index	0.5207	0.0054
SEED_WATER_CONTENT	Flower, sepal length	0.5024	0.0076
FRESH_WEIGHT	Flower, sepal length	0.5	0.0079
NUMBER_OF_SEEDS	Seed, number	0.4982	0.0082
TOTAL_PODS	Cotyledon mass	−0.4775	0.0118
TOTAL_PODS	Cotyledon length	−0.4772	0.0118
POD_WEIGHT	Flower, sepal length	0.4768	0.0119
POD_LENGTH	Flower, sepal length	0.4736	0.0126
DRY_WEIGHT	Flower, sepal length	0.4711	0.0131
SEED_WATER_CONTENT	Flower, ligule width	0.4701	0.0134
POD_INDEX	Flower, sepal length	−0.4679	0.0139
DRY_WEIGHT	Cotyledon length	0.4659	0.0143
POD_INDEX	Cotyledon length	−0.4658	0.0143
TOTAL_PODS	Wet bean mass	−0.4644	0.0147
POD_LENGTH	Fruit, width	0.4623	0.0152
POD_INDEX	Flower, ligule width	−0.4605	0.0156
FRESH_WEIGHT	Flower, ligule width	0.4548	0.0171
DRY_WEIGHT	Flower, ligule width	0.4543	0.0173
FRESH_WEIGHT	Fruit, length	0.4473	0.0193
SEED_WATER_CONTENT	Fruit, length	0.4461	0.0197
Cotyledon length to width ratio	Seed, number	0.445	0.02
INFECTION_RATE	Cotyledon length	0.444	0.0203
INFECTION_RATE	Cotyledon mass	0.4435	0.0205
YIELD	Flower, ovule number	0.4432	0.0206
INFECTION_RATE	Cotyledon width	0.437	0.0227
POD_WEIGHT	Fruit, length	0.4296	0.0253
FRESH_WEIGHT	Wet bean mass	0.4225	0.0281
POD_LENGTH	Flower, ligule width	0.4199	0.0292
YIELD	Seed, number	0.4157	0.031
FRESH_WEIGHT	Pod index	−0.4109	0.0333
POD_WEIGHT	Flower, ligule width	0.4066	0.0353
SEED_WATER_CONTENT	Pod index	−0.406	0.0356
POD_WEIGHT	Wet bean mass	0.4054	0.0359
POD_WEIGHT	Pod index	−0.4029	0.0372
POD_WIDTH	Flower, sepal length	0.3877	0.0457

**Fig. 5:**
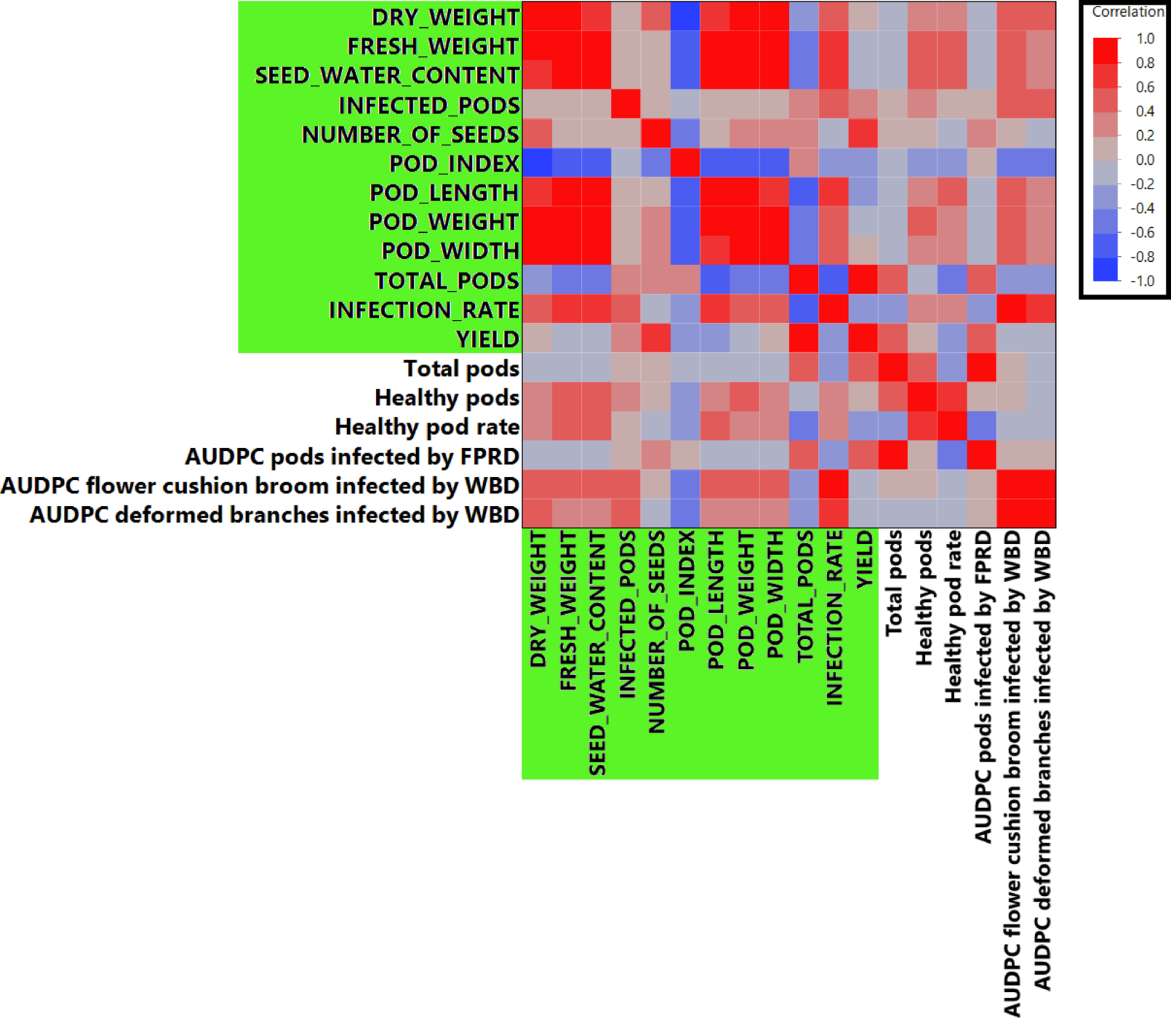
Comparative analysis using Pearson’s correlations of traits between the TARS evaluation dataset and the Agrosavia collection dataset for 20 overlapping cacao accessions. Traits from the TARS study are highlighted with a green background on the axes. Pearson correlation coefficients (*r*) are visualized by color intensity (red for positive, blue for negative). Detailed correlation coefficients and *p*-values are provided in Supplementary Data 1

Pearson’s correlation analysis was conducted between numerical traits from the TARS study and those reported by Osorio-Guarín et al. [7] for 20 overlapping accessions evaluated in Colombia. The correlation matrix is shown in Fig. 5, with TARS traits highlighted in green (full details in Supplementary Data 1). Notably, ‘Infection rate (TARS)’, primarily reflecting black pod rot incidence caused by *Phytophthora* spp. in Puerto Rico, showed strong positive correlations with disease metrics from the Agrosavia study. Specifically, it was correlated with ‘AUDPC flower cushion broom infected by WBD’ (*r* = 0.86, *p* < 0.0001) and ‘AUDPC deformed branches infected by WBD’ (*r* = 0.72, *p* = 0.0016). This result indicates that accessions susceptible to black pod rot in Puerto Rico were also susceptible to Witches’ Broom Disease (WBD) in Colombia. Other significant correlations included ‘Fresh seed weight (TARS)’ with ‘AUDPC flower cushion broom infected by WBD’ (*r* = 0.56, *p* = 0.025), ‘Total number of pods (TARS)’ with ‘AUDPC pods infected by WBD’ (*r* = 0.50, *p* = 0.024), and ‘Pod weight (TARS)’ with ‘AUDPC flower cushion broom infected by WBD’ (*r* = 0.56, *p* = 0.024).

### Machine learning models predict Cacao yield from TARS phenotypic traits

To assess the utility of phenotypic traits for predicting cacao yield in the PR study, a model screening approach employing various ML algorithms was conducted using JMP Pro 17 with 5-fold cross-validation. The predictive performance and key contributing traits were evaluated in an iterative manner.

Initially, when all other measured phenotypic traits were used as predictors for ‘Yield’, a Neural Boosted model demonstrated superior performance, achieving an average R^2^ of 0.9985 across the 5 validation folds. Other models, such as Fit Stepwise (average R^2^ = 0.9571), Generalized Regression Lasso (average R^2^ = 0.9539), Boosted Tree (average R^2^ = 0.9332), and Fit Least Squares (average R^2^ = 0.9234), also performed well, while Bootstrap Forest, SVM-RBF, Decision Tree, and KNN models had average R^2^ values below 0.90. The best individual validation model from this iteration was a Neural Boosted model (Fold 1, R^2^ = 0.9994). For this model, ‘Total pods’ was the most influential predictor, with a total effect of 0.763, followed by ‘Pod index’ (total effect = 0.213) and ‘Dry weight’ (total effect = 0.054). Other traits had total effects of less than 0.002 (details in Supplementary Data 1). This near-perfect R-squared value is expected, as several key predictors (e.g., ‘Total pods’, ‘Pod index’) are mathematical components of the target variable ‘Yield’. The value of this analysis, therefore, lies not in prediction per se, but in confirming and ranking the hierarchy of these components through our iterative approach.

In a second iteration, ‘Total pods’ was removed from the set of predictor variables. The Neural Boosted algorithm remained the top-performing model, with an average validation R-squared of 0.9935 across 5 folds. The next best models were SVM-RBF (average R^2^ = 0.76) and Generalized Regression Lasso (average R^2^ = 0.7371). The best individual validation model in this iteration was a Neural Boosted model (Fold 3, R^2^ = 0.999). The primary predictor identified by this model was ‘Infection rate’, with a total effect of 0.722. Other important predictors included ‘Fresh seed weight’ (total effect = 0.469), ‘Infected pods’ (total effect = 0.274), ‘Seed water content’ (total effect = 0.259), and ‘Pod weight’ (total effect = 0.224) (Supplementary Data 1).

Finally, with both ‘Total pods’ and ‘Infection rate’ excluded as predictors, the Neural Boosted model still provided the best average predictive performance across 5 folds, though the average R-squared decreased to 0.5214. Bootstrap Forest (average R^2^ = 0.3606) and Fit Stepwise (average R^2^ = 0.3417) were the next best. The best individual validation model was again a Neural Boosted model (Fold 1), achieving an R^2^ of 0.6884. In this constrained model, ‘Pod weight’ emerged as the most significant predictor (total effect = 0.543), followed by ‘Infected pods’ (total effect = 0.217), ‘Number of seeds’ (total effect = 0.118), ‘Pod index’ (total effect = 0.091), and ‘Pod width’ (total effect = 0.09) (Supplementary Data 1). These iterative modeling experiments highlight the strong predictive capacity of phenotypic traits for cacao yield and reveal a hierarchy of trait importance.

### Marker-trait association identifies loci on multiple chromosomes linked to key horticultural traits

**Fig. 6:**
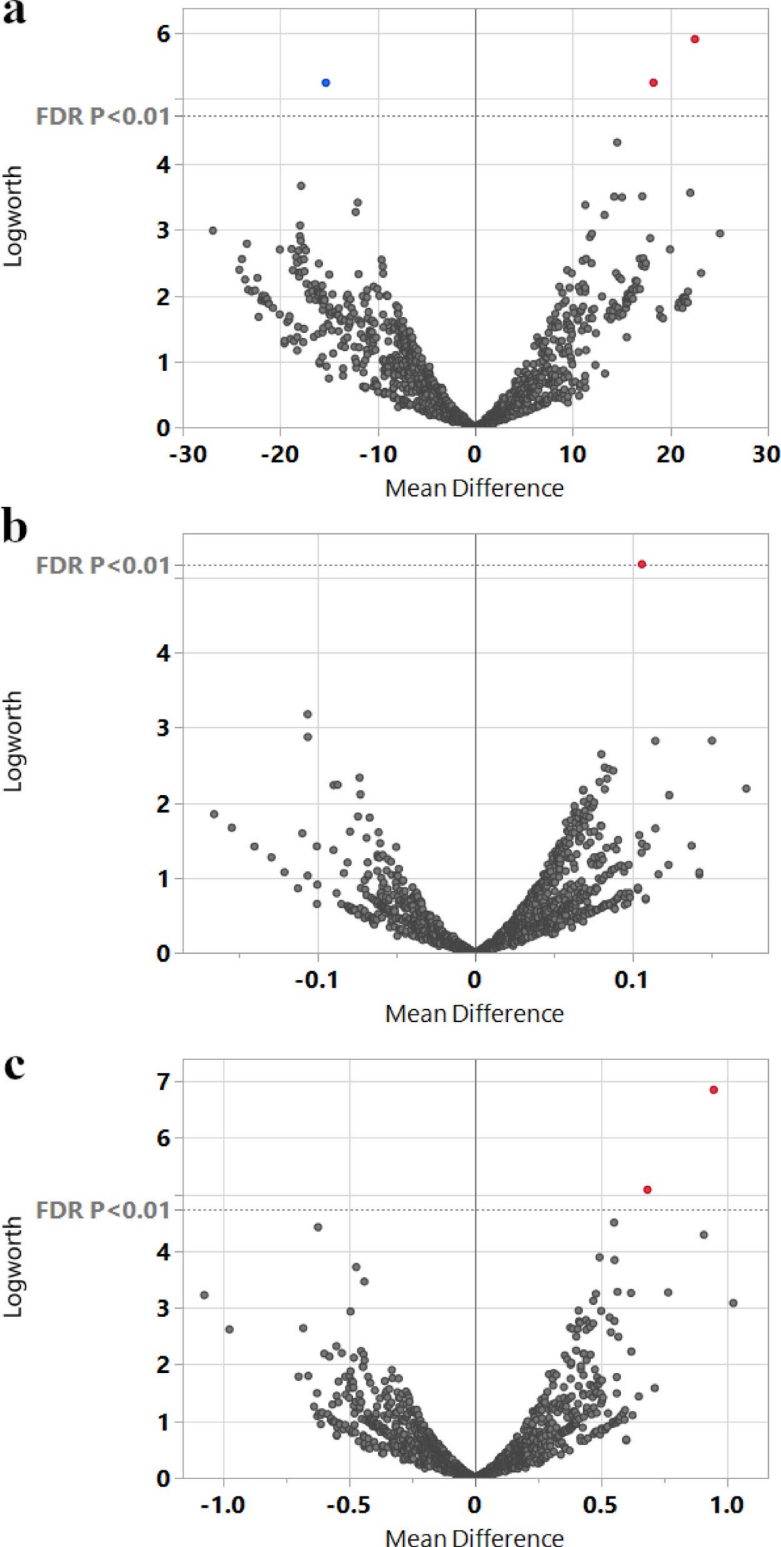
Volcano plots illustrating marker associations with key horticultural traits from the TARS cacao collection. Response screening analysis using genetic markers from the ICGT study as predictors for traits in 28 common accessions. In each plot, the x-axis represents the mean difference associated with each marker, the y-axis represents the Logworth score, and each point represents a single marker. The horizontal dashed line indicates the significance threshold (FDR *p* < 0.01). Points colored red or blue highlight the markers that surpassed this threshold. (a) Volcano plot for the ‘Total pods’ trait, highlighting three significant markers (the locus TcSNP475, and SNPs TcSNP428 and TcSNP154). (b) Volcano plot for the ‘Infection rate’ trait, highlighting one significant SNP (TcSNP508). (c) Volcano plot for the ‘Yield’ trait, highlighting two significant markers (the locus TcSNP475 and SNP TcSNP483)

To identify genetic markers associated with phenotypic variation observed in the accessions evaluated in TARS, SNP data from Bekele et al. [22] were integrated with the TARS phenotypic dataset for 27 common accessions. Utilizing 671 SNPs, a response screening analysis was conducted in JMP Pro 17. This analysis identified several SNPs significantly associated with key horticultural traits after applying a FDR correction (*p* < 0.01). For ‘Total pods’, three significant markers passed the significance threshold (Fig. 6a). These included the genetic marker TcSNP475, located within a putative zinc finger stress-associated protein gene (*Tc05_t008610*); TcSNP428, associated with a magnesium-protoporphyrin cyclase gene (*Tc05v2_g011210*); and TcSNP154, linked to a xyloglucan endotransglucosylase/hydrolase protein gene (*Tc01v2_g018540*). For ‘Infection rate’, a single marker, TcSNP508, was found to be significant (Fig. 6b) and is associated with a cysteine proteinase 15 A gene (*Tc08v2_g002970*). For ‘Yield’, two SNPs were significantly associated (Fig. 6c). One was again the genetic locus TcSNP475, reinforcing its link to overall productivity. The other significant marker was TcSNP483, located within a gene for an ATP synthase subunit (*Tc03v2_g019660*). No other SNPs reached this level of genome-wide significance for the other horticultural traits tested with this FDR threshold. The repeated association of the genetic marker TcSNP475 on chromosome 5 with both ‘Total pods’ and ‘Yield’ is particularly noteworthy. This locus is located within the gene *Tc05_t008610*, annotated as encoding a “Zinc finger A20 and AN1 domain-containing stress-associated protein 4”. This finding strongly points to this genomic region as a potential quantitative trait locus (QTL) influencing yield components in this subset of cacao germplasm, with a candidate gene potentially involved in stress response pathways.

## Discussion

### Phenotypic diversity and trait architecture in the TARS collection

The comprehensive analysis of a diverse cacao collection (173 accessions) evaluated at TARS has provided significant insights into its phenotypic architecture and the intricate interplay of key horticultural traits; such foundational knowledge is essential for targeted crop improvement strategies [33]. The considerable phenotypic diversity within the collection was structured using multivariate analyses, including PCA (Fig. 1) and hierarchical clustering (Fig. S3), which revealed distinct phenotypic groupings and trait relationships. This approach is instrumental for breeders in identifying phenotypically superior and diverse individuals to maximize heterosis or combine valuable traits [34, 35]. The strong positive correlation observed between ‘Total pods’ and ‘Yield’ (*r* = 0.83, Fig. 2) across the entire collection reinforces the direct selection pressure on pod number as a primary yield driver, a well-established relationship in cacao [34, 36]. Conversely, the strong negative correlation between ‘Dry seed weight’ (total per pod) and ‘Pod index’ (*r* = − 0.95, Fig. 2) highlights a key metric for processing efficiency. The finding further explains this relationship, as a higher ‘Number of seeds’ per pod was positively correlated with the total ‘Dry seed weight’ (*r* = 0.45) and, consequently, was also negatively correlated with ‘Pod index’ (*r* = − 0.50). In essence, pods with more seeds yield a greater total mass of dry beans, meaning fewer of these productive pods are required to make one kilogram [36]. These intra-collection interdependencies point to key trait relationships and potential physiological trade-offs that breeders must navigate [33, 35, 36]. For instance, ‘Total pods’ was negatively correlated with both ‘Pod weight’ (*r* = − 0.48) and ‘Dry seed weight’ (*r* = − 0.32). This reflects a classic yield component compensation, a phenomenon noted to occur in cacao where inverse relationships between components like pod number and bean weight are observed [37]. This compensation mechanism means that plants producing a higher number of pods often do so at the expense of individual pod and seed size, resulting in a lower seed mass per pod [37]. Therefore, aiming for an optimal balance between these components, rather than maximizing a single one, is a more effective strategy for improving overall plant productivity and resource allocation [33]. A key practical outcome of this germplasm characterization is the identification of accessions with superior performance for specific traits of interest to breeding programs. The full dataset, from which top-performing accessions for traits such as high yield, low pod index, and low disease incidence can be identified, is provided in Supplementary Data 1. This detailed information provides a valuable resource for breeders seeking to select parents with specific desirable characteristics for developing new, improved cacao varieties.

### Trait stability and environmental interactions revealed by comparative analysis

Beyond the general trait interdependencies, the analysis of the 27-accession subset provided a direct link between genetic background and horticultural traits in the PR environment. ANOVA revealed significant mean differences in key traits like Yield, Total Pods, Dry Seed Weight, and Pod Index among the K = 7 genetic clusters defined by Bekele et al. [22] (Fig. 4). The correlation analysis provided further nuance, showing how the degree of association to these clusters correlated with specific traits (Fig. 3). For instance, a stronger affiliation with the ‘AMAZ, IMC’ genetic cluster was significantly correlated with higher Yield (*r* = 0.50) and an increased Number of Seeds (*r* = 0.59), demonstrating that the quantitative degree of ancestry can predict performance for specific desirable traits.

The analysis of this 27-accession subset al.so allowed for a valuable, albeit complex, cross-environmental perspective through multi-environment evaluations, which are essential for dissecting genotype performance and identifying stable or specifically adapted lines where G×E interactions often complicate breeding progress. The comparison between the TARS and ICGT datasets revealed several key relationships (Table 1, Fig. S4). Evidence for trait stability was observed, with a significant positive correlation for ‘Number of seeds’ between the two sites (*ρ* = 0.50, *p* = 0.0082). For example, accessions such as IMC 67, SPA7, and SPA9, ranked highly for ‘Number of seeds’ in both the TARS and ICGT evaluations, identifying them as potentially stable genetic resources for this trait. The analysis also highlighted consistent biological trade-offs, such as the negative correlation between ‘Total pods (TARS)’ and ‘Cotyledon mass (ICGT)’ (*ρ*= −0.48,*p* = 0.0118). Furthermore, an intriguing positive correlation was found between ‘Infection rate (TARS)’ and ‘Cotyledon mass (ICGT)’ (*ρ* = 0.44, *p* = 0.0205), which may suggest a trade-off between resource investment in seed size and disease defense mechanisms. The strong positive correlation between ‘Infection rate (TARS)’, primarily caused by black pod rot due to *Phytophthora* spp., and WBD metrics from the Osorio-Guarín et al. study [7] is a particularly robust and intriguing finding. This correlation between two different diseases in two different environments suggests that certain cacao accessions possess a consistent genotypic response to disease pressure. This could be indicative of broad-spectrum disease resistance mechanisms, where resistance genes (R-genes) or quantitative trait loci confer tolerance to multiple, distinct pathogens, a highly valuable trait for breeding programs.

### Novel marker-trait associations and candidate genes for cacao improvement

The integration of SNP data with TARS phenotypic data for 28 common accessions identified multiple significant marker-trait associations, a significant expansion of the original findings (Fig. 6). A key molecular finding was the association of the genetic marker TcSNP475 on chromosome 5 with both ‘Total pods’ and ‘Yield’. This dual association reinforces the importance of this locus as a QTL for productivity. This locus is located within the gene *Tc05_t008610*, annotated as a “Zinc finger A20 and AN1 domain-containing stress-associated protein 4”. This gene family, often termed Stress-Associated Proteins (SAPs), is increasingly recognized for its pivotal roles in mediating plant responses to a wide array of abiotic (e.g., salinity, drought, cold, heat) and biotic stresses, as well as influencing growth and development across diverse plant species [38–41]. Indeed, functional studies have demonstrated that plant SAPs, such as those from rice, can confer enhanced tolerance to multiple environmental stresses when overexpressed [42, 43]. Consequently, SAPs are considered important candidate genes for strategies aimed at enhancing stress tolerance in crops, which can, in turn, significantly impact productivity and yield stability, particularly under suboptimal environmental conditions [40–42]. The A20/AN1 zinc finger domains are characteristic features of these proteins, with the A20 domain, for instance, implicated in mediating protein-protein interactions crucial for stress signaling pathways [38, 42]. Beyond this key locus, the analysis revealed other novel associations with biologically relevant candidate genes. For ‘Total pods’, an association was found with TcSNP154, linked to a xyloglucan endotransglucosylase/hydrolase (XTH) gene (*Tc01v2_g018540*). XTHs are key enzymes that modify the primary cell wall by restructuring xyloglucan tethers, a process critical for cell expansion during fruit growth and for the textural changes that occur during ripening [44, 45]. For ‘Infection rate’, a significant association was identified with TcSNP508, located within a cysteine protease gene (*Tc08v2_g002970*). Cysteine proteases are integral components of plant innate immunity, contributing to defense responses against pathogens through various mechanisms, including the regulation of programmed cell death to limit infection [46, 47]. Finally, a second marker for ‘Yield’, TcSNP483, was associated with a gene encoding an ATP synthase subunit (*Tc03v2_g019660*). The abundance and activity of ATP synthase are directly linked to photosynthetic capacity and CO₂ assimilation, making it a fundamental control point for overall plant growth and productivity [48, 49]. These findings offer a suite of new, tangible targets for further research and hold potential for future application in MAS (Marker-Assisted Selection) for cacao improvement [8].

### Predictive modeling as a tool for cacao breeding

The primary value of the machine learning models in this study was not prediction per se, but rather to quantify the hierarchy of trait importance through an iterative modeling strategy. The iterative removal of top predictors (‘Total pods’, then ‘Infection rate’) in our models revealed a clear hierarchy of trait importance and the robustness of this modeling strategy. Although the R-squared decreased as key predictors were removed (to 0.9935 without ‘Total pods’, and to 0.5214 without both ‘Total pods’ and ‘Infection rate’), the models still captured a significant portion of yield variance. This ability to identify and rank the importance of various predictors is a key strength of ML applications in agriculture [11, 16]. Our findings indicate that while ‘Total pods’ is paramount for yield prediction in this dataset, other factors such as disease pressure (represented by ‘Infection rate’) and inherent pod characteristics (‘Pod weight’, ‘Number of seeds’) play substantial secondary and tertiary roles. For breeders, this implies that even if direct pod counts are unavailable or variable, other phenotypic assessments, informed by such predictive models, can still provide valuable yield estimations. This is particularly relevant for identifying productive clones at early stages or streamlining selection processes [8, 11]. The identification of ‘Infection rate’ as a strong secondary predictor in our models particularly underscores the critical impact of disease management on realizing yield potential, a crucial consideration for sustainable cacao production systems [8].

### Limitations of the study

Despite these promising findings, several limitations of this study must be acknowledged. The core phenotypic data characterizing the 173 accessions evaluated at TARS for this study originate from a single location. This inherently limits the ability to dissect G×E interactions for these specific accessions without further trials, as a comprehensive understanding and exploitation of G×E (and G×E×Management) interactions often require multi-environment trials or advanced modeling approaches to define the target population of environments and reduce interaction effects [14, 15]. The marker-trait association study, while identifying several significant markers, was conducted on a relatively small subset of 28 common accessions and utilized a marker panel developed for a different primary population, which, as we determined, contained named genetic loci in addition to single-nucleotide polymorphisms [22]. These factors, including sample size and the characteristics of the SNP panel in relation to the study population, can affect the statistical power for association detection and the broader applicability of the identified marker [50]. The comparative phenotypic analyses, while insightful, also contend with data generated under different environmental conditions, management practices, and non-standardized phenotyping protocols, which can confound direct comparisons. Notably, the significant differences in tree age between the collections (~ 6–10 years at TARS vs. ~18–20 years at Agrosavia, and a variable range at ICGT) are a potential confounding factor, particularly for age-dependent traits like yield.

Furthermore, while the identities of the TARS accessions were genetically verified, our comparative analyses relied on the accession identities as reported in the source publications; therefore, any underlying errors in accession identity (homonymy or synonymy) in those collections could be a confounding factor in the cross-environmental comparisons. It is also noteworthy that the analysis using over 8,000 SNP markers from the Agrosavia collection did not yield any significant marker-trait associations. This could be due to the small number of common accessions (*n* = 20) or strong genotype-by-environment (G×E) interactions masking the effects of these specific markers in the Puerto Rican environment. In addition, the ML models, despite their high predictive accuracy on this dataset, require validation on independent cacao populations to ascertain their generalizability. Finally, the study is largely observational and correlational; while we identify associations and potential candidate genes, establishing causal relationships will necessitate dedicated functional genomics studies. Additionally, it must be noted that all accessions in the TARS evaluation were grafted onto a single rootstock type (‘Amelonado’). While this ensured uniformity within the trial, the specific influence of this rootstock on scion performance is a confounding factor in the cross-environmental comparisons.

### Future directions

Future research should prioritize the validation of the suite of markers identified in this study (e.g., TcSNP475, TcSNP483, TcSNP508) across a broader range of cacao germplasm and diverse environments. Fine-mapping the associated QTL regions and conducting functional characterization studies (e.g., gene expression analysis, transgenic approaches) on their respective candidate genes, including the zinc finger stress-associated protein (*Tc05_t008610*), the cysteine proteinase, and the ATP synthase subunit, are crucial steps to confirm their roles in cacao yield, disease response, and overall productivity while developing new cacao germplasm. Building on the existing, though limited, SNP and SSR marker sets for this collection, future research should focus on expanding the genomic data for more accessions. Employing higher-density methods, such as genotyping-by-sequencing, would be a critical step to enable more comprehensive GWAS and genomic prediction studies than is currently possible. Multi-location trials incorporating promising accessions identified here would be invaluable for assessing G×E interactions and identifying broadly adapted, high-yielding, and resilient genotypes. Investigating the mechanistic basis for observed cross-study trait correlations, such as the link between infection rate and seed mass, could also yield novel insights for integrated pest and disease management strategies.

## Conclusion

This research delivers a comprehensive phenotypic and correlational landscape of a diverse cacao collection (173 accessions) evaluated at TARS, revealing its significant genetic diversity and the intricate interplay of key horticultural traits. Notably, ML models demonstrated high predictive accuracy for yield using these phenotypic data, successfully identifying a hierarchy of influential traits. Furthermore, comparative analyses with established regional datasets elucidated the clear impact of environmental specificity on cacao performance, while also identifying conserved trait relationships, such as for seed number, and key biological trade-offs across environments. A critical molecular outcome of this study was the identification of multiple SNPs significantly associated with key horticultural traits, including ‘Total pods’, ‘Infection rate’, and ‘Yield’. The most noteworthy finding was the association of a locus on chromosome 5, marked by TcSNP475, with both ‘Total pods’ and ‘Yield’. This marker is located within a putative zinc finger stress-associated protein gene, strongly pointing to this region as a key QTL for cacao productivity. While the inherent limitations of the study are acknowledged, these integrated findings collectively offer valuable resources and novel insights for advancing cacao genetic improvement. This research equips cacao breeders with new predictive tools and a set of candidate markers for more efficient selection. Based on our predictive modeling, we recommend that breeding programs with limited resources prioritize the evaluation of ‘Total pods’, ‘Infection rate’, and ‘Pod weight’, as these were identified as the most influential predictors of yield. Ultimately, this research provides molecular geneticists with a basis for future investigations into the fundamental mechanisms of productivity and stress tolerance in this vital global crop.

## Supplementary Information

Below is the link to the electronic supplementary material.

Supplementary Material 1

Supplementary Material 2

## Data Availability

All data generated or analyzed during this study are included in this published article and its supplementary information files are available from public repositories: The primary phenotypic dataset evaluated in Puerto Rico is publicly available through the USDA-ARS Germplasm Resources Information Network (GRIN)-Global database under the group name “CACAO.MAYAGUEZ.2007-2011” (method ID 495345). The phenotypic and genotypic data from the International Cocoa Genebank, Trinidad (ICGT), sourced from Bekele et al. (2022), are publicly available (https://journals.plos.org/plosone/article? id=10.1371/journal.pone.0260907). The phenotypic and genotypic data from the Agrosavia collection are available within the original publication by Osorio-Guarín et al. (2020) (https://academic.oup.com/g3journal/article/10/5/1713/6026284). Detailed results for the statistical analyses conducted in this study, including complete correlation matrices and machine learning model performance metrics, are available in the supplementary materials associated with this article.
