# Supplementary material for: Integrated phenotypic analysis, predictive modeling, and identification of novel trait-associated loci in a diverse Theobroma cacao collection
Source: BMC Plant Biol. 2025 Aug 9;25:1050. doi: 10.1186/s12870-025-07128-y (PMC12335022; doi:10.1186/s12870-025-07128-y)
Supplement: Supplementary file 2 — Supplementary Material 2 [file 12870_2025_7128_MOESM2_ESM.docx]

**Supplementary Figures**


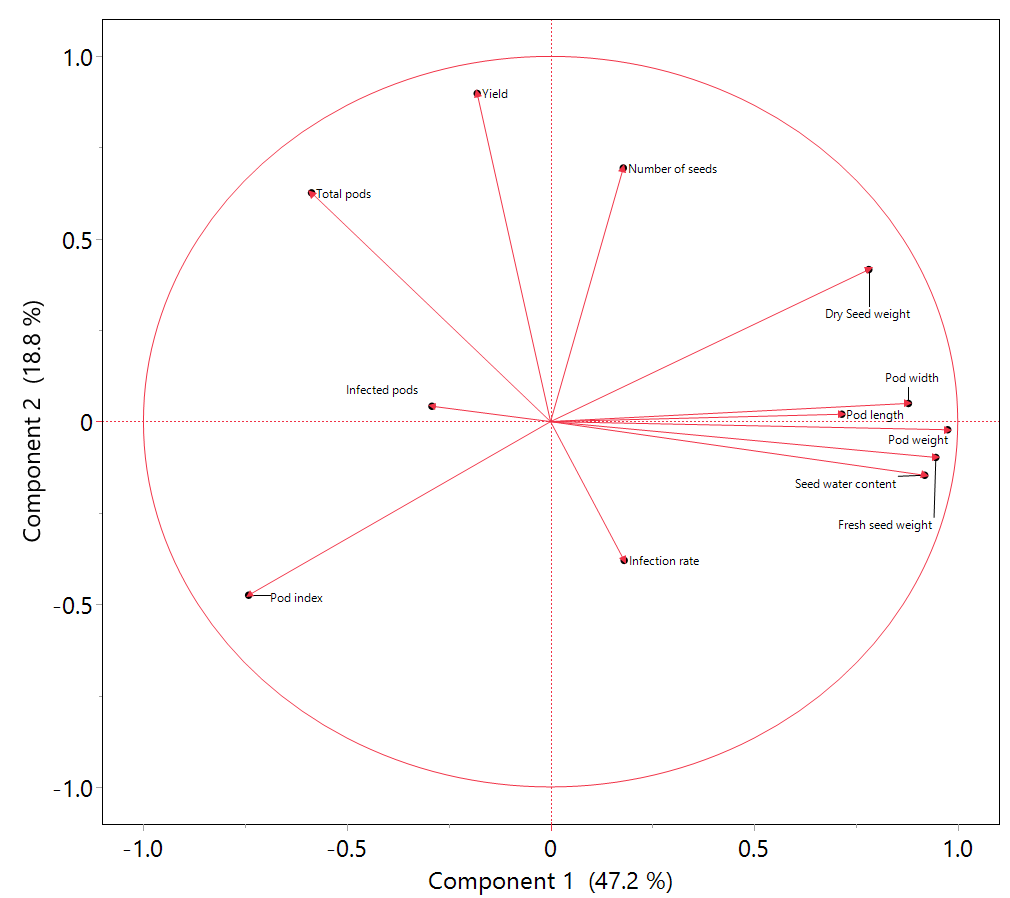


**Fig. S1** **Loading plot from the PCA of phenotypic traits.** The plot shows the contribution of each measured horticultural trait to the first two principal components (PC1 and PC2). Vectors indicate the direction and strength of each trait's influence on the principal components. Traits positioned further from the origin have a stronger influence. This plot corresponds to the PCA shown in Fig. 1.


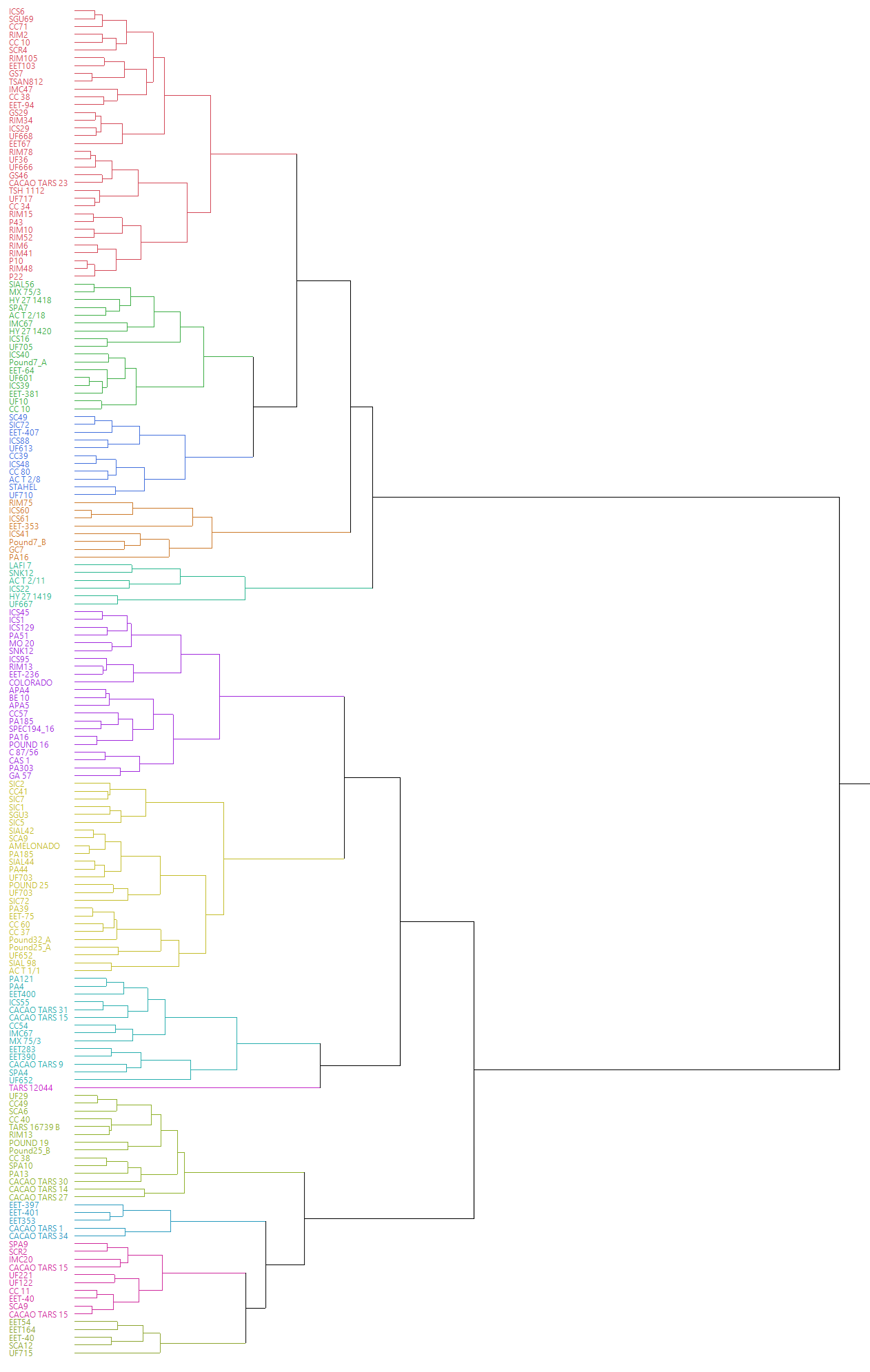


**Fig. S2 Hierarchical clustering of cacao accessions.** Dendrogram showing hierarchical clustering of 173 cacao accessions using Ward's method based on all measured phenotypic traits. Different colors in the dendrogram branches delineate putative clusters.


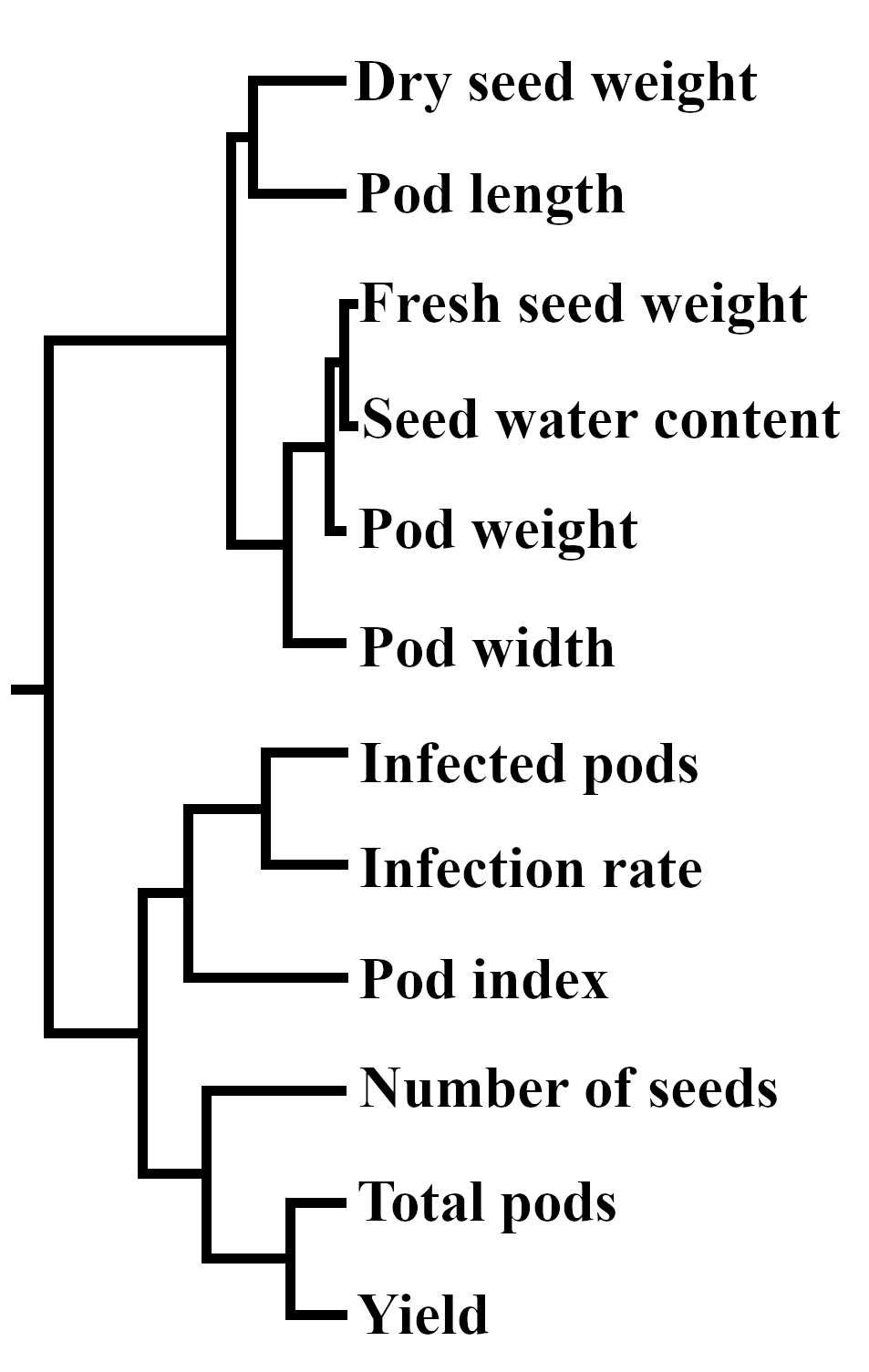


**Fig. S3 Hierarchical clustering of cacao horticultural traits.** Dendrogram illustrating the hierarchical clustering of the horticultural traits themselves, based on their relationships across the 173 accessions.


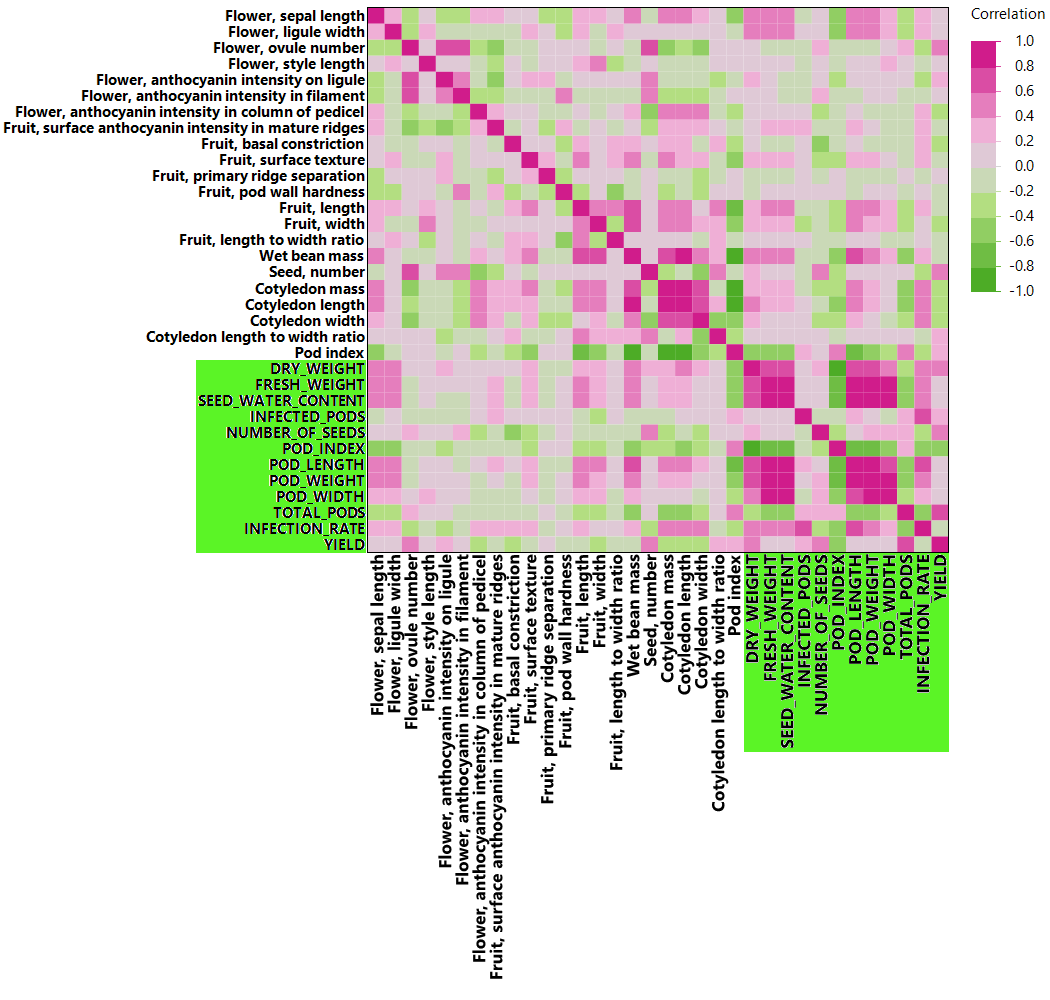


**Fig. S4 Comparative analysis using Spearman’s rank correlation of traits between the TARS evaluation dataset and the ICGT (Trinidad) dataset for 27 overlapping cacao accessions.** Spearman's rank correlation matrix. Traits from the TARS study are highlighted with a green background on the axes. Correlation coefficients (*ρ*) are visualized by color intensity (red for positive, green for negative) according to the scale.
